# Information-Geometric Approach for a One-Sided Truncated Exponential Family

**DOI:** 10.3390/e25050769

**Published:** 2023-05-08

**Authors:** Masaki Yoshioka, Fuyuhiko Tanaka

**Affiliations:** 1 Graduate School of Engineering Science, Osaka University, Osaka 560-8531, Japan; 2 Center for Education in Liberal Arts and Sciences, Osaka University, Osaka 560-0043, Japan; ftanaka.celas@osaka-u.ac.jp

**Keywords:** truncated exponential family, non-regular model, common scale parameter, alpha-parallel prior, information geometry, statistical manifold, 62B11, 62F12

## Abstract

In information geometry, there has been extensive research on the deep connections between differential geometric structures, such as the Fisher metric and the *α*-connection, and the statistical theory for statistical models satisfying regularity conditions. However, the study of information geometry for non-regular statistical models is insufficient, and a one-sided truncated exponential family (oTEF) is one example of these models. In this paper, based on the asymptotic properties of maximum likelihood estimators, we provide a Riemannian metric for the oTEF. Furthermore, we demonstrate that the oTEF has an *α* = 1 parallel prior distribution and that the scalar curvature of a certain submodel, including the Pareto family, is a negative constant.

## 1. Introduction

Information geometry is the study of the structure of statistical models using differential geometry. From the standpoint of geometry, a statistical model consisting of a collection of parameterized probability distributions can be regarded as a manifold. Then, when the statistical model satisfies a certain regularity condition, Chentsov’s theorem leads to a natural differential geometric structure [1]. This natural differential geometric structure consists of the Riemann metric defined from the Fisher information matrix and a one-parameter family of affine connections, called the Fisher metric and the *α*-connection, respectively.

Information geometry of regular models has been studied for a long time, and deep relationships between the geometric structures and statistical models have been revealed (Amari [2] and Amari and Nagaoka [3]).

However, the geometric properties of statistical models which do not satisfy regularity conditions are not sufficiently investigated. One reason is that Chentsov’s theorem cannot be applied to non-regular models; thus, a natural geometric structure is not established. For example, in regular models, it is possible to define the Fisher information matrix in two forms, but in statistical models where the support of the probability density function depends on the parameters, they do not coincide. In previous work, Amari [4] discussed the relationship between the Finsler geometry and non-regular models especially for the translation family.

In the present study, we discuss information geometry for a one-sided truncated exponential family (oTEF) [5], a typical non-regular model. An oTEF is a statistical model with two parameters: the natural parameter *θ* and the truncation parameter *γ*. The support of the probability density function depends on the truncation parameter *γ*, which is what makes such a model non-regular. However, similar to the exponential family, the derivatives of the log-likelihood function, moments, and KL divergence can be explicitly described. Additionally, an oTEF model is a model containing many practical examples, such as the Pareto distribution family, truncated normal distribution family, and truncated exponential distribution family. The statistical properties of the oTEF have been studied for a long time, particularly in the area of parameter estimation theory (Bar-Lev [5], Akahira [6], and Akahira [7]). Recently, Nielsen [8] studied the relationship between the Kullback–Leibler divergence and the Bhattacharyya distance in the oTEF. Shemyakin [9] explored the Hellinger information matrix and its application to noninformative priors for multiparameter non-regular cases, which includes the oTEF, within the framework of Bayesian statistics.

The family of Pareto distributions, a type of oTEF, is a topic of study in information geometry as well (Rylov [10], Peng et al. [11], Li et al. [12], and Sun et al. [13]). As a result, it is known that the Pareto model has constant curvature with respect to the Levi-Civita connection. However, it is impossible to introduce a natural geometric structure, so the geometric structure of the Pareto distribution family has been formally defined based on regular models. Consequently, the consistency with statistical theory remains to be determined. Moreover, multiple geometric structures exist depending on the regular model expressions used as references. For example, Peng et al. [11], Rylov [10], and Sun et al. [13] all define the geometric structure of the Pareto distribution family based on the Fisher metric and *α*-connection, but these structures are different. Therefore, the question of which geometric structure should be used remains unresolved.

The results of the present study consist of two theorems: one regarding the *α*-parallel prior distribution and another concerning curvature. First of all, by comparing the relationship between the Fisher metric and the asymptotic behavior of the maximum likelihood estimators in regular models with those in the oTEF, we define the Riemannian metric for the oTEF. Additionally, we use a formal definition for the *α*-connection, similar to Sun et al. [13]. Under this geometric structure, we demonstrate two geometric properties. The first property is that the oTEF always has an *α*-parallel prior distribution with *α* = 1. The *α*-parallel prior distribution is a type of noninformative prior distribution used in Bayesian statistics and is an information-geometric extension of Jeffreys prior distribution. Although the *α*-parallel prior distribution with *α* = 1 always exists, this is not true for other *α* values. Such a phenomenon does not occur in regular models, highlighting the non-regularity. The second research result is related to the curvature of a submodel of the oTEF. We consider a submodel of the oTEF that includes common scale Pareto distribution families [14] and common location exponential distribution families [15] and demonstrate that its scalar curvature is constant. This result, stating that the scalar curvature of this submodel is constant, corresponds to a higher-dimensional version of the fact that the Pareto model has constant curvature [10].

The remainder of this paper is organized as follows. In Section 2, we review the geometric structure of regular statistical models and one-sided truncated exponential families, providing an overview of the relevant background. In Section 3, we provide a Riemannian metric and affine connection in the oTEF, giving the family a differential geometric structure. Under the geometric structure, we study the *α*-parallel prior to the oTEF in Section 4. Finally, Section 5 gives our derivation of the curvature for a submodel of the oTEF.

## 2. Preliminaries

### 2.1. Statistical Manifold

In this section, we review basic definitions and notation of information geometry for regular statistical models. See work by Amari [2] for more details.

Let $P=\left\{P_{\theta }:\theta \in \Theta \right\}$ be a family of distributions $P_{\theta }$ on a sample space χ parametrized by $\theta =(\theta^{1},\ldots ,\theta^{n})$, where Θ is an open subset of $R^{n}$. We treat the statistical model P as an *n*-dimensional manifold. In this case, parameter θ takes the role of coordinates of the manifold P. Furthermore, we assume that parameter θ and distribution $P_{\theta }$ have a one-to-one correspondence. Furthermore, suppose that all distributions in P are absolutely continuous with respect to a dominating measure μ. We denote the probability density function of distribution $P_{\theta }$ with respect to μ as p(x,θ).

In information geometry, we assume certain conditions on P.

**Definition** **1** (Regular statistical models)**.** *A statistical model P is said to be regular if it satisfies the following conditions.*

*1.*  *The support of the density function*
  (1) $$\begin{array}{c} \mathrm{supp}p=\bar{\left\{x\in \chi :p(x,\theta )>0\right\}} \end{array}$$
  *is independent of* θ∈Θ*.*
*2.*  *For all* x∈χ*, the function* θ↦p(x,θ) *is* $C^{\infty }$ *on* Θ*.*
*3.*  *Let $l\left(x,\theta \right)=\log p\left(x,\theta \right)$. The elements of $\{\partial_{1}l,\ldots ,\partial_{n}l$} are linearly independent as functions on χ.*
*4.*  *For any functions in the following, partial differentiation $\partial_{i}$ and integration with respect to the measure μ are interchangeable as*
(2) $$\begin{array}{c} \int_{\chi }\partial_{i}f\left(x,\theta \right)d\mu \left(x\right)=\partial_{i}\int_{\chi }f\left(x,\theta \right)d\mu \left(x\right). \end{array}$$

The above four conditions are referred to as *regularity conditions*.

We will treat non-regular statistical models in later sections. In preparation, this subsection discusses the geometric structure of regular statistical models.

Next, we introduce the Fisher metric and *α*-connection. Let P be a regular statistical model and *X* be a random variable distributed according to $P_{\theta }$. We usually use the following definition of the Riemannian metric and affine connections on P.

**Definition** **2** (Fisher metric)**.** *The Fisher metric is the Riemannian metric on P defined by*

(3) $$\begin{array}{cc} g_{ij}^{F} & =E_{\theta }\left[\partial_{i}l\left(X,\theta \right)\partial_{j}l\left(X,\theta \right)\right], \end{array}$$

*for i,j=1,…,n. Here, the symbol $E_{}\left[\cdot \right]$ denotes the expectation with respect to the observation X.*

**Definition** **3** (*α*-connection)**.** *The α-connection is an affine connection defined by the coefficients*

(4) $$\begin{array}{cc} {\overset{\left(\alpha \right)}{\Gamma }}_{ij,k} & =E_{}\left[\partial_{i}\partial_{j}l\left(X,\theta \right)\partial_{k}l\left(X,\theta \right)\right]+\frac{1-\alpha }{2}E_{}\left[\partial_{i}l\left(X,\theta \right)\partial_{j}l\left(X,\theta \right)\partial_{k}l\left(X,\theta \right)\right]\;, \end{array}$$

*for i,j,k=1,…,n and α∈R on the coordinate system θ.*

If P satisfies the regularity conditions, the Fisher metric and the *α*-connection have several properties. For instance, there is a formula [16] convenient for calculating the Fisher metric:

(5) $$\begin{array}{cc} g_{ij}^{F} & =-E_{}\left[\partial_{i}\partial_{j}l\left(x,\theta \right)\right]. \end{array}$$

Furthermore, the *α*-connection and the −*α*-connection are *dual*. In other words,

(6) $$\begin{array}{c} A\langle B,C\rangle =\langle {\overset{\left(\alpha \right)}{\nabla }}_{A}\;B,C\rangle +\langle B,{\overset{\left(-\alpha \right)}{\nabla }}_{A}\;C\rangle \end{array}$$

holds for all tangent vectors *A*, *B*, and *C*, where $\langle \cdot ,\cdot \rangle$ denotes the inner product given by the Fisher metric. In particular, the 0-connection is self-dual. Furthermore, the 0-connection corresponds to the *Levi-Civita connection*, defined by the coefficients

(7) $$\begin{array}{cc} {\overset{g}{\Gamma }}_{ij,k} & =\frac{1}{2}\left(\partial_{i}g_{jk}^{F}+\partial_{j}g_{ki}^{F}-\partial_{k}g_{ij}^{F}\right). \end{array}$$

### 2.2. Prior Distributions and Volume Elements

Next, we introduce volume elements on a regular statistical model and its relation to Bayesian statistics [17].

In Bayesian statistics, for a given statistical model P, we need a probability distribution over the model parameter space, which is called a *prior distribution* or simply a *prior*. We often denote a prior density as π. (π(θ)≥0 and $\int_{\Theta }\pi \left(\theta \right)d\theta =1.$)

A volume element on an *n*-dimensional oriented model manifold corresponds to a prior density function over the parameter space ($\theta \in \Theta \subset R^{n}$) in a one-to-one manner. For a prior π(θ), its corresponding volume element ω is an *n*-form (differential form of degree *n*) and is written as

(8) $$\begin{array}{c} \omega =\pi \left(\theta \right)d\theta^{1}\wedge \cdots \wedge d\theta^{n} \end{array}$$

in the local coordinates.

For example, in the two-dimensional Euclidian space (n=2), the volume element is given by ω=dx∧dy in the Cartesian coordinates (x,y). In the polar coordinates (r,ψ), it is written as ω=rdr∧dψ.

Now, let us explain noninformative priors (see, e.g., work by Robert [18] for details). If we have specific information on the parameter in advance, then the prior should reflect it, in which case it is often called a *subjective prior*. If not, we adopt a certain criterion and use a prior obtained through the criterion. Such priors are called *noninformative priors* or *objective priors*. In particular, the Jeffreys prior, which is given by $\sqrt{\det(g)}$, is the standard noninformative prior [19].

In information geometry, the Jeffreys prior is regarded as a 0-parallel volume element and was extended to an *α*-parallel volume element by Takeuchi and Amari [17]. The extensions are called *α*-parallel priors. They showed that the geometrical properties of regular models are deeply related to the existence of *α*-parallel priors.

When a suitable geometric structure is defined on a non-regular statistical model, it is interesting to see the relationship between the geometrical properties and the existence of *α*-parallel priors. Later sections will discuss this topic. In preparation, in this subsection, we briefly summarize some definitions and facts of *α*-parallel priors for regular models.

To define *α*-parallel priors, we introduce a geometric property of affine connections.

**Definition** **4** (Equiaffine)**.** *Let* P *be an n-dimensional manifold with an affine connection induced by a covariant derivative* ∇*.*

*An affine connection* ∇ *is equiaffine if there exists a volume element ω such that*

(9) $$\begin{array}{c} \nabla \omega =0 \end{array}$$

*holds everywhere in* P*. Furthermore, such a volume element ω is said to be a parallel volume element with respect to* ∇*.*

The necessary and sufficient condition for an affine connection ∇ to be equiaffine is described by its curvature. The following proposition holds for a manifold with an affine connection ∇. Let ${R_{ijk}}^{l}$ be the components of the Riemannian curvature tensor [3] of ∇, defined as

(10) $$\begin{array}{c} {R_{ijk}}^{l}=\partial_{i}{\Gamma_{jk}}^{l}-\partial_{i}{\Gamma_{ik}}^{l}+{\Gamma_{im}}^{l}{\Gamma_{jk}}^{m}-{\Gamma_{jm}}^{l}{\Gamma_{ik}}^{m},\;\left(i,j,k,l=1,\ldots ,n\right) \end{array}$$

where ${\Gamma_{ij}}^{k}$ denotes the connection coefficients of ∇.

**Proposition** **1**  (Nomizu and Sasaki [20])**.** *The following conditions are equivalent:*

- ∇ *is equiaffine,*
- $R_{ijk}{\;}^{k}=0$*,*

*where $R_{ijk}{\;}^{k}=\sum_{k=1}^{n}R_{ijk}{\;}^{k}$.*

The Levi-Civita connection is always equiaffine (Nomizu and Sasaki [20]).

Returning to the statistical model, we define the α-parallel prior distribution. Let P be an *n*-dimensional regular statistical manifold. It is shown that $\overset{\left(\alpha \right)}{\nabla }$ is equiaffine for some α∈R\{0} if and only if it is equiaffine for all α∈R. Such a statistical manifold P is said to be *statistically equiaffine*. In a statistically equiaffine manifold, we may represent the α-parallel volume element $\omega^{\left(\alpha \right)}$ as

(11) $$\begin{array}{c} \omega^{\left(\alpha \right)}=\pi^{\left(\alpha \right)}\left(\theta \right)d\theta^{1}\wedge \cdots \wedge d\theta^{n} \end{array}$$

for the coordinates θ, where $\pi^{\left(\alpha \right)}\in C^{\infty }\left(P\right)$. We take $\pi^{\left(\alpha \right)}\left(\theta \right)$ as a prior distribution on the parameter space Θ.

**Definition** **5** (α-parallel prior)**.** *In a statistically equiaffine manifold, for fixed α∈R, we call the above form of $\pi^{\left(\alpha \right)}$ an α-parallel prior.*

Since the 0-connection (Levi-Civita connection) is equiaffine, there always exists a 0-parallel prior, known as the Jeffreys prior.

Takeuchi and Amari [17] give a necessary and sufficient condition for *α*-parallel priors to exist.

**Proposition** **2**  (Takeuchi and Amari [17])**.** *For a model manifold P, if*

(12) $$\begin{array}{c} \partial_{i}T_{jk}{\;}^{k}-\partial_{j}T_{ik}{\;}^{k}=0\;\left(i,j=1,\ldots ,n\right), \end{array}$$

*then the α-parallel prior exists for any α∈R. Otherwise, only the 0-parallel prior exists.*

### 2.3. One-Sided Truncated Exponential Family

In this section, we introduce a one-sided truncated exponential family, which is a typical non-regular model.

**Definition** **6**  (One-sided Truncated Exponential Family (Bar-Lev [5]))**.** *A one-sided truncated exponential family of distributions (oTEF) is a family* $P=\left\{P_{\theta ,\gamma }:\theta \in \Theta ,\gamma \in \left(I_{1},I_{2}\right)\right\}$ of distributions $P_{\theta ,\gamma }$ *with the density functions*

(13) $$\begin{array}{cc} p(x,\theta ,\gamma ) & =\exp\left\{\sum_{i=1}^{n}\theta^{i}F_{i}\left(x\right)+C\left(x\right)-\psi \left(\theta ,\gamma \right)\right\}\cdot {𝟙}_{\left[\gamma ,I_{2}\right)}\left(x\right)\;\left(x\in \left(I_{1},I_{2}\right)\right) \end{array}$$

*with respect to the Lebesgue measure. Here, $-\infty \leq I_{1}<I_{2}\leq \infty$ are known parameters, C is a continuous function, and $F_{1},\ldots ,F_{n}$ are absolutely continuous with $dF_{1}\left(x\right)/dx,\ldots ,dF_{n}\left(x\right)/dx\neq 0$ over the interval $\left(I_{1},I_{2}\right)$.*

We say $\theta =\left(\theta^{1},\cdots ,\theta^{n}\right)$ is the natural parameter and γ is the truncation parameter. We also denote the interval $\left(I_{1},I_{2}\right)$ as *I*.

In oTEF P, the support of the density function depends on the truncation parameter γ. Then, the oTEF does not satisfy the regularity conditions. An oTEF differs from an exponential family at this point. The density of an exponential family has a support that is independent of the parameters.

Furthermore, this truncation parameter γ does not allow for the interchange of partial differentiation $\partial_{\gamma }$ and integration with respect to the Lebesgue measure. For instance, this means

(14) $$\begin{array}{c} E_{}\left[\partial_{\gamma }l\left(X,\theta ,\gamma \right)\right]=-\partial_{\gamma }\psi \left(\theta ,\gamma \right) \end{array}$$

instead of $E_{}\left[\partial_{\gamma }l\left(X,\theta ,\gamma \right)\right]=0$.

Here, we introduce two properties of the function ψ.

First, the partial derivative $-\partial_{\gamma }\psi \left(\theta ,\gamma \right)$ coincides with ${\left.p(x,\theta ,\gamma )\right|}_{x=\gamma }$ and is always positive. This fact can be verified as follows. Since *p* is a probability density function,

(15) $$\begin{array}{c} \exp\left\{\psi \left(\theta ,\gamma \right)\right\}=\int_{\gamma }^{I_{2}}\exp\left\{\sum_{i=1}^{n}\theta^{i}F_{i}\left(x\right)+C\left(x\right)\right\}dx. \end{array}$$

Therefore, by differentiating both sides with respect to γ, we obtain

(16) $$\begin{array}{c} \partial_{\gamma }\psi \left(\theta ,\gamma \right)\exp\left\{\psi \left(\theta ,\gamma \right)\right\}=-\exp\{\sum_{i=1}^{n}\theta^{i}F_{i}\left(\gamma \right)+C\left(\gamma \right)\} \end{array}$$

and

(17) $$\begin{array}{c} -\partial_{\gamma }\psi \left(\theta ,\gamma \right)={\left.p(x,\theta ,\gamma )\right|}_{x=\gamma }>0. \end{array}$$

Second, the following lemma holds.

**Lemma** **1.** *For the function ψ(θ,γ) in (13), there always exists θ and γ such that $\partial_{i}\partial_{\gamma }\psi \left(\theta ,\gamma \right)\neq 0$.*

**Proof.**  Suppose that $\partial_{\gamma }\partial_{i}\psi \left(\theta ,\gamma \right)$ is always 0. Under this assumption, we will show that $\partial_{i}p\left(x,\theta ,\gamma \right)\equiv 0$, leading to a contradiction that θ is not a parameter.

If $\partial_{\gamma }\partial_{i}\psi \left(\theta ,\gamma \right)\equiv 0$, the function ψ(θ,γ) can be expressed as

(18) $$\begin{array}{c} \psi \left(\theta ,\gamma \right)=\psi_{1}\left(\theta \right)+\psi_{2}\left(\gamma \right), \end{array}$$

where $\psi_{1}\in C^{\infty }\left(\Theta \right),\;\psi_{2}\in C^{\infty }\left(I\right)$.

Then,

(19) $$\begin{array}{c} \exp\left\{\psi_{1}\left(\theta \right)+\psi_{2}\left(\gamma \right)\right\}=\int_{\gamma }^{I_{2}}\exp\left\{C\left(x\right)+\sum \theta^{i}F_{i}\left(x\right)\right\}dx. \end{array}$$

Differentiating both sides with respect to γ, we obtain

(20) $$\begin{array}{c} \psi_{2}^{\prime }\left(\gamma \right)e^{\psi_{1}\left(\theta \right)+\psi_{2}\left(\gamma \right)}=-\exp\{C\left(\gamma \right)+\sum \theta^{i}F_{i}\left(\gamma \right)\}, \end{array}$$

(21) $$\begin{array}{c} \log\left\{-\psi_{2}^{\prime }\left(\gamma \right)e^{\psi_{2}\left(\gamma \right)-C\left(\gamma \right)}\right\}+\psi_{1}\left(\theta \right)=\sum \theta^{i}F_{i}\left(\gamma \right). \end{array}$$

Further differentiating both sides with respect to $\theta^{i}$, we have

(22) $$\begin{array}{c} \partial_{i}\psi_{1}\left(\theta \right)=F_{i}\left(\gamma \right) \end{array}$$

and all $F_{i}$ are constants.

Therefore,

(23) $$\begin{array}{cc} \partial_{i}p\left(x,\theta ,\gamma \right) & =p\left(x,\theta ,\gamma \right)\partial_{i}\{C\left(x\right)+\sum \theta^{i}F_{i}-\psi_{1}\left(\theta \right)-\psi_{2}\left(\gamma \right)\} \end{array}$$

(24) $$\begin{array}{c} =p\left(x,\theta ,\gamma \right)\{F_{i}-\partial_{i}\psi_{1}\left(\theta \right)\} \end{array}$$

(25) $$\begin{array}{c} =0. \end{array}$$

□

The family of Pareto distributions is an example of the oTEF. Pareto distributions have the following density functions,

(26) $$\begin{array}{cc} p(x,\theta ,\gamma ) & =\frac{\theta \gamma^{\theta }}{x^{\theta +1}}\cdot {𝟙}_{\left[\gamma ,\infty \right)}\left(x\right) \end{array}$$

(27) $$\begin{array}{cc} \; & \;=\exp\left(-\left(1+\theta \right)\log x+\log\theta +\theta \log\gamma \right)\cdot {𝟙}_{\left[\gamma ,\infty \right)}\left(x\right)\; \end{array}$$

with the natural parameter θ∈(0,∞) and the truncation parameter γ∈(0,∞). This family is used to describe various natural and social phenomena [21].

To discuss geometric structures in subsequent sections, we focus on the asymptotic behavior of maximum likelihood estimators in the oTEF. Our discussion of these estimators follows previous works. Consider random variables $X_{1},\ldots ,X_{N}$ independent and identically distributed according to $P_{\theta ,\gamma }$, and let $X_{\left(1\right)}\leq ,\cdots \leq ,X_{\left(N\right)}$ be the order statistics of the sample. Let $\hat{\theta }$ and $\hat{\gamma }$ denote the maximum likelihood estimators for θ and γ, respectively. Bar-Lev [5] showed the existence and uniqueness of $\hat{\theta }$ and $\hat{\gamma }$. Here, $\hat{\gamma }=X_{\left(1\right)}$ and $\hat{\theta }$ is a root of the maximum likelihood equation $\partial_{i}l\left(X,\hat{\theta },\hat{\gamma }\right)=0$ for i=1,…,n.

The first-order asymptotic variances of $\hat{\theta }$ and $\hat{\gamma }$ are given by

(28) $$\begin{array}{cc} V_{}\left[\hat{\theta }\right] & =\frac{1}{N\partial_{i}\partial_{j}\psi \left(\theta ,\gamma \right)}+O\left(\frac{1}{N^{2}}\right), \end{array}$$

(29) $$\begin{array}{cc} V_{}\left[\hat{\gamma }\right] & =\frac{1}{N^{2}{\left\{\partial_{\gamma }\psi \left(\theta ,\gamma \right)\right\}}^{2}}+O\left(\frac{1}{N^{3}}\right), \end{array}$$

(30) $$\begin{array}{cc} {\mathrm{Cov}}_{}\left[\hat{\theta },\hat{\gamma }\right] & =O\left(\frac{1}{N^{3}}\right). \end{array}$$

These are essential to our argument in the next section. Additionally, Akahira [6] and Akahira and Ohyauchi [22] obtained the second-order asymptotic loss.

## 3. Geometric Structure on One-Sided Exponential Families

This section gives definitions of a geometric structure of the oTEF. We take P as an (n+1)-dimensional manifold with coordinates $\left(\theta^{1},\cdots ,\theta^{n},\gamma \right)$. Since P does not satisfy regularity conditions, its geometric structure is not determined in a natural way.

### 3.1. Riemannian Metric in the oTEF

In this subsection, we define a Riemannian metric on oTEF P as follows.

**Definition** **7** (Riemannian metric in the oTEF)**.** *Let*$P=\left\{P_{\theta ,\gamma }:\theta \in \Theta ,\gamma \in I\right\}$ *belong to the oTEF. The Riemannian metric of the oTEF is defined by*

(31) $$\begin{array}{cc} g_{ij} & =E_{}\left[\partial_{i}l\left(x,\theta ,\gamma \right)\partial_{j}l\left(x,\theta ,\gamma \right)\right], \end{array}$$

(32) $$\begin{array}{cc} g_{i\gamma } & =0, \end{array}$$

(33) $$\begin{array}{cc} g_{\gamma \gamma } & ={\left\{\partial_{\gamma }\psi \left(\theta ,\gamma \right)\right\}}^{2} \end{array}$$

*for i,j=1,…,n. This metric is also represented as*

(34) $$\begin{array}{c} \left(g\right)=\left(\begin{array}{cccc} g_{11} & \cdots & g_{1n} & 0\\ \vdots & \ddots & \vdots & \vdots \\ g_{n1} & \cdots & g_{nn} & 0\\ 0 & \cdots & 0 & {\left\{\partial_{\gamma }\psi \left(\theta ,\gamma \right)\right\}}^{2} \end{array}\right). \end{array}$$

Note that $g_{ij}$ can be expressed in terms of the function ψ as follows:

(35) $$\begin{array}{c} g_{ij}=-E_{}\left[\partial_{i}\partial_{j}l\left(x,\theta ,\gamma \right)\right]=\partial_{i}\partial_{j}\psi \left(\theta ,\gamma \right). \end{array}$$

This is similar to the case of exponential family distributions.

We will now explain how we came to the above definition.

Consider an exponential family $E_{\gamma }=\left\{P_{\theta ,\gamma }:\theta \in \Theta \right\}$ for γ∈I and a statistical model $F_{\theta }=\left\{P_{\theta ,\gamma }:\gamma \in I\right\}$ for θ∈Θ. $E_{\gamma }$ is an *n*-dimensional submanifold of P obtained by fixing the truncation parameter γ and satisfies the regularity conditions. Additionally, $F_{\theta }$ is a one-dimensional submanifold of P obtained by fixing the natural parameter θ. Since $E_{\gamma }$ is regular, the Riemannian metric on $E_{\gamma }$ should be the Fisher metric defined in Definition 2. This idea induces the components $g_{ij}$ to be the components of the Fisher metric. The remaining task is to define $g_{i\gamma }$, the inner products of $\partial_{i}$ and $\partial_{\gamma }$, and $g_{\gamma \gamma }$, the metric on $F_{\theta }$.

In this context, we review the statistical interpretation of the Fisher metric in regular models. Let $P_{0}$ be a regular model with parameters $\theta \in R^{n}$. As mentioned in Section 2.1, the Fisher metric is a Riemannian metric defined by the Fisher information matrix. Expanding the variance of the maximum likelihood estimator $\hat{\theta }$, we have

(36) $$\begin{array}{c} V_{}\left[\hat{\theta }\right]=\frac{1}{N}{\left(g_{ij}^{F}\right)}^{-1}+O\left(\frac{1}{N^{2}}\right), \end{array}$$

where the first-order coefficient corresponds to ${\left(g_{ij}^{F}\right)}^{-1}$.

On the other hand, in the oTEF, the Riemannian metric is determined from the coefficient of the variance of the maximum likelihood estimator. As shown in Section 2.3, the variances of $\hat{\theta }$ and $\hat{\gamma }$ are expressed as

(37) $$\begin{array}{cc} V_{}\left[\hat{\theta }\right] & =\frac{1}{N}{\left(g_{ij}\right)}^{-1}+O\left(\frac{1}{N^{2}}\right), \end{array}$$

(38) $$\begin{array}{cc} V_{}\left[\hat{\gamma }\right] & =\frac{1}{N^{2}{\left\{\partial_{\gamma }\psi \left(\theta ,\gamma \right)\right\}}^{2}}+O\left(\frac{1}{N^{3}}\right), \end{array}$$

(39) $$\begin{array}{cc} {\mathrm{Cov}}_{}\left[\hat{\theta },\hat{\gamma }\right] & =O\left(\frac{1}{N^{3}}\right), \end{array}$$

where $\left(g_{ij}\right)$ is the matrix ${\left(g_{ij}\right)}_{i,j=1}^{n}$. Then, similarly to the Fisher information matrix, ${\left\{\partial_{\gamma }\psi \left(\theta ,\gamma \right)\right\}}^{2}$ appears as the reciprocal of the first-term coefficient. From this, the Riemannian metric of $F_{\theta }$ is defined as

(40) $$\begin{array}{c} g_{\gamma \gamma }={\left\{\partial_{\gamma }\psi \left(\theta ,\gamma \right)\right\}}^{2}. \end{array}$$

Furthermore, it should be noted that ${\mathrm{Cov}}_{}\left[\hat{\theta },\hat{\gamma }\right]$ is negligible up to $O\left(\frac{1}{N^{2}}\right)$. Moreover, whether θ is known does not affect the first-order term of $V_{}\left[\hat{\theta }\right]$ [22]. This is also true for the estimation of θ [23]. Based on these facts, we assume that $\partial_{i}$ and $\partial_{\gamma }$ are orthogonal, and define

(41) $$\begin{array}{c} g_{i\gamma }=0\;\left(i=1,\ldots ,n\right). \end{array}$$

As a result, the Riemannian metric in Definition 7 is obtained.

This Riemannian metric is equal to the formally defined Fisher metric on the oTEF. In other words, the equality

(42) $$\begin{array}{c} g_{ab}=E_{}\left[\partial_{a}l\left(X,\theta ,\gamma \right)\partial_{b}l\left(X,\theta ,\gamma \right)\right] \end{array}$$

holds for a,b=1,…,n,γ. The right-hand side is the same as the definition of the Fisher metric on regular statistical models.

However, the Riemann metric does not satisfy the equation

(43) $$\begin{array}{cc} g_{a\gamma } & =-E_{}\left[\partial_{a}\partial_{\gamma }l\left(x,\theta ,\gamma \right)\right]\;\left(a=1,\ldots ,n,\gamma \right). \end{array}$$

For i=1,…,n, we have

(44) $$\begin{array}{cc} g_{i\gamma } & =0, \end{array}$$

(45) $$\begin{array}{cc} E_{}\left[\partial_{i}\partial_{\gamma }l\left(x,\theta ,\gamma \right)\right] & =\partial_{i}\partial_{\gamma }\psi \left(\theta ,\gamma \right), \end{array}$$

but $\partial_{i}\partial_{\gamma }\psi \left(\theta ,\gamma \right)\neq 0$ for some θ and γ, by Lemma 1. This is influenced by the fact that it does not satisfy the regularity conditions.

### 3.2. Affine Connections in the oTEF

Next, we define an affine connection in the oTEF. In this study, we adopt two types of connections: the Levi-Civita connection and the α-connection.

The first affine connection is the *Levi-Civita connection*:

(46) $$\begin{array}{cc} {\overset{g}{\Gamma }}_{ab,c} & =\partial_{a}g_{bc}+\partial_{b}g_{ca}-\partial_{c}g_{ab} \end{array}$$

introduced from the Riemannian metric. Of course, this Levi-Civita connection is also a metric and self-dual, the same as in the regular case. Rylov [10] and Li et al. [12] previously adopted this same affine connection.

Second, we define an affine connection in the oTEF as an analogy for the α-connection in the regular statistical model defined in Definition 3.

**Definition** **8.** *For a given α∈R, the α-connection in the oTEF is defined by the connection coefficients*

(47) $$\begin{array}{c} {\overset{\left(\alpha \right)}{\Gamma }}_{ab,c}\left(\theta ,\gamma \right)=E_{}\left[\partial_{a}\partial_{b}l\partial_{c}l\right]+\frac{1-\alpha }{2}E_{}\left[\partial_{a}l\partial_{b}l\partial_{c}l\right]\;\left(a,b,c=1,\ldots ,n,\gamma \right), \end{array}$$

*where l=l(X,θ,γ) is a log-likelihood function.*

The above definition is obtained by substituting the oTEF probability density function into Equation (4) for the *α*-connection in the regular model. In particular, for the Pareto distribution family, it coincides with the *α*-connection given by Sun et al. [13] (see the equation in their paper). Note that, the log-likelihood function is not differentiable with respect to *γ* at γ=x. We calculate the expectation over the interval $\left(\gamma ,I_{2}\right)$ instead of $\left[\gamma ,I_{2}\right)$.

This α-connection is torsion-free,

(48) $$\begin{array}{c} {\overset{\left(\alpha \right)}{\Gamma }}_{ab,c}={\overset{\left(\alpha \right)}{\Gamma }}_{ba,c}. \end{array}$$

The connection coefficients for the α-connection are given by

(49) $$\begin{array}{cc} {\overset{\left(\alpha \right)}{\Gamma }}_{ij,k} & =\frac{1-\alpha }{2}\partial_{i}\partial_{j}\partial_{k}\psi , \end{array}$$

(50) $$\begin{array}{cc} {\overset{\left(\alpha \right)}{\Gamma }}_{ij,\gamma } & =\frac{1+\alpha }{2}\partial_{i}\partial_{j}\psi \partial_{\gamma }\psi , \end{array}$$

(51) $$\begin{array}{cc} {\overset{\left(\alpha \right)}{\Gamma }}_{i\gamma ,j} & =-\frac{1-\alpha }{2}\partial_{i}\partial_{j}\psi \partial_{\gamma }\psi , \end{array}$$

(52) $$\begin{array}{cc} {\overset{\left(\alpha \right)}{\Gamma }}_{i\gamma ,\gamma } & =\partial_{i}\partial_{\gamma }\psi \partial_{\gamma }\psi , \end{array}$$

(53) $$\begin{array}{cc} {\overset{\left(\alpha \right)}{\Gamma }}_{\gamma \gamma ,i} & =0, \end{array}$$

(54) $$\begin{array}{cc} {\overset{\left(\alpha \right)}{\Gamma }}_{\gamma \gamma ,\gamma } & =\partial_{\gamma }\partial_{\gamma }\psi \partial_{\gamma }\psi -\frac{1-\alpha }{2}{\left(\partial_{\gamma }\psi \right)}^{3}, \end{array}$$

where ψ=ψ(θ,γ).

The α-connection in the oTEF differs from the one in the regular model in several aspects.

First, the 0-connection does not correspond to the Levi-Civita connection. This is due to the inability to express the α-connection coefficients in the following form:

(55) $$\begin{array}{c} {\overset{\left(\alpha \right)}{\Gamma }}_{abc}(\theta ,\gamma )={\overset{g}{\Gamma }}_{abc}(\theta ,\gamma )-\frac{\alpha }{2}E_{}\left[\partial_{a}l\partial_{b}l\partial_{c}l\right]. \end{array}$$

This transformation involves an interchange of the order of differentiation and integration, which is one of the regularity conditions. Therefore, in the non-regular model oTEF, the two sides do not match.

Additionally, the dual connection of the α-connection in the oTEF does not become the −α-connection. This can be verified as follows.

The partial derivative $\partial_{\gamma }g_{i\gamma }$ and connection coefficients ${\overset{\left(\alpha \right)}{\Gamma }}_{\gamma i,\gamma }$ and ${\overset{\left(-\alpha \right)}{\Gamma }}_{\gamma \gamma ,i}$ are given by

(56) $$\begin{array}{cc} \partial_{\gamma }g_{\gamma i} & =0, \end{array}$$

(57) $$\begin{array}{cc} {\overset{\left(\alpha \right)}{\Gamma }}_{\gamma i,\gamma } & =\partial_{i}\partial_{\gamma }\psi \left(\theta ,\gamma \right)\partial_{\gamma }\psi \left(\theta ,\gamma \right), \end{array}$$

(58) $$\begin{array}{cc} {\overset{\left(-\alpha \right)}{\Gamma }}_{\gamma \gamma ,i} & =0. \end{array}$$

Thus,

(59) $$\begin{array}{cc} {\overset{\left(\alpha \right)}{\Gamma }}_{\gamma i,\gamma }+{\overset{\left(-\alpha \right)}{\Gamma }}_{\gamma \gamma ,i} & =\partial_{\gamma }\partial_{i}\psi \left(\theta ,\gamma \right)\partial_{\gamma }\psi \left(\theta ,\gamma \right). \end{array}$$

Therefore, since $\partial_{\gamma }\partial_{i}\psi \left(\theta ,\gamma \right)\neq 0$ and $\partial_{\gamma }\psi \left(\theta ,\gamma \right)<0$, the duality does not hold.

## 4. Existence of an *α*-Parallel Prior in the oTEF

In this section, as part of investigating the properties of the *α*-connection in the oTEF, we deal with the *α*-parallel priors. We show that there exists an *α*-parallel prior distribution for *α* = 1.

The existence of *α*-parallel priors depends on the geometric properties of statistical models, and they are not guaranteed to exist in general. Therefore, it is necessary to investigate the existence of *α*-parallel priors in the case of the oTEF. Note that the Levi-Civita connection is always equiaffine, and it is known to have the Jeffreys prior as a parallel prior distribution. Therefore, we do not deal with it in this section.

In the oTEF, attention is needed to be paid to the conditions for the existence of the *α*-parallel prior distributions. In the case of regular models, Proposition 2 provides a necessary and sufficient condition for the existence of *α*-parallel priors. Sun et al. [13] revealed that the Pareto distribution family does not satisfy this condition and claims that it does not have *α*-parallel priors. However, the above deduction is incorrect, since Proposition 2 does not hold for an oTEF distribution. In proof [17] of Proposition 2, the connection coefficients of the *α*-connection are written in the same form as (55) by the Levi-Civita connection and the cubic tensor $T_{ijk}=E_{}\left[\partial_{i}l\partial_{j}l\partial_{k}l\right]$. However, this form cannot represent the *α*-connection in Definition 8. Then, in the oTEF, Proposition 2 does not confirm that the equation

(60) $$\begin{array}{c} \partial_{a}T_{bc}{\;}^{c}-\partial_{b}T_{ac}{\;}^{c}=0\;\left(a,b=1,\ldots ,n,\gamma \right) \end{array}$$

is a necessary and sufficient condition for the existence of *α*-parallel priors.

Therefore, in this study, we use the necessary and sufficient conditions for general affine connections to investigate the existence of the *α*-parallel prior.

The following theorem reveals the existence of *α*-parallel priors in the oTEF.

**Theorem** **1.** *Consider P as belonging to the oTEF with densities of the form as those in Definition 13 with the natural parameter θ∈Θ and the truncation parameter γ. If α=1, then the connection $\overset{\left(\alpha \right)}{\nabla }$ is equiaffine and there exists a one-parallel prior. Moreover, this one-parallel prior $\pi^{\left(1\right)}$ can be represented as*

(61) $$\begin{array}{c} \pi^{\left(1\right)}\left(\theta ,\gamma \right)\propto -\partial_{\gamma }\psi \left(\theta ,\gamma \right) \end{array}$$

*for θ∈Θ,γ∈I.*

**Proof.**  First, we prove that $\overset{\left(1\right)}{\nabla }$ is equiaffine. By Proposition 1, the necessary and sufficient condition for $\overset{\left(\alpha \right)}{\nabla }$ to be equiaffine is ${\overset{\left(\alpha \right)}{R}}_{abc}{}^{c}=0$ for a,b=1,…,n,γ everywhere in P, where $\overset{\left(\alpha \right)}{R}$ denotes *α*-Riemannian curvature tensors. This condition can be represented as $\partial_{a}{\overset{\left(\alpha \right)}{\Gamma }}_{bc}{}^{c}=\partial_{b}{\overset{\left(-\alpha \right)}{\Gamma }}_{ac}{}^{c},$ since

(62) $$\begin{array}{cc} {\overset{\left(\alpha \right)}{R}}_{abc}{}^{c} & =\sum_{c,d=c}\{\partial_{a}{\overset{\left(\alpha \right)}{\Gamma }}_{bc}{}^{d}-\partial_{b}{\overset{\left(\alpha \right)}{\Gamma }}_{ac}{}^{d}+{\overset{\left(\alpha \right)}{\Gamma }}_{ae}{}^{d}{\overset{\left(\alpha \right)}{\Gamma }}_{bc}{}^{e}-{\overset{\left(\alpha \right)}{\Gamma }}_{be}{}^{d}{\overset{\left(\alpha \right)}{\Gamma }}_{ac}{}^{e}\} \end{array}$$

(63) $$\begin{array}{c} =\partial_{a}{\overset{\left(\alpha \right)}{\Gamma }}_{bc}{}^{c}-\partial_{b}{\overset{\left(\alpha \right)}{\Gamma }}_{ac}{}^{c}+{\overset{\left(\alpha \right)}{\Gamma }}_{ae}{}^{c}{\overset{\left(\alpha \right)}{\Gamma }}_{bc}{}^{e}-{\overset{\left(\alpha \right)}{\Gamma }}_{be}{}^{c}{\overset{\left(\alpha \right)}{\Gamma }}_{ac}{}^{e} \end{array}$$

(64) $$\begin{array}{cc} \; & =\partial_{a}{\overset{\left(\alpha \right)}{\Gamma }}_{bc}{}^{c}-\partial_{b}{\overset{\left(\alpha \right)}{\Gamma }}_{ac}{}^{c}. \end{array}$$

By Definition 7, we have

(65) $$\begin{array}{cc} {\overset{\left(\alpha \right)}{\Gamma }}_{ja}{}^{a} & =\frac{1-\alpha }{2}\partial_{j}\log\left(\det(g_{kl})\right)+\partial_{j}\log\left(-\partial_{\gamma }\psi \right), \end{array}$$

(66) $$\begin{array}{cc} {\overset{\left(\alpha \right)}{\Gamma }}_{\gamma a}{}^{a} & =-\frac{\left(n+1\right)\left(1-\alpha \right)}{2}\partial_{\gamma }\psi +\partial_{\gamma }\log\left(-\partial_{\gamma }\psi \right). \end{array}$$

Thus,

(67) $$\begin{array}{cc} \partial_{i}{\overset{\left(\alpha \right)}{\Gamma }}_{ja}{}^{a}-\partial_{j}{\overset{\left(\alpha \right)}{\Gamma }}_{ia}{}^{a} & =0, \end{array}$$

(68) $$\begin{array}{cc} \partial_{\gamma }{\overset{\left(\alpha \right)}{\Gamma }}_{ia}{}^{a}-\partial_{i}{\overset{\left(\alpha \right)}{\Gamma }}_{\gamma a}{}^{a} & =\frac{1-\alpha }{2}\partial_{i}\partial_{\gamma }\log\left(\det(g_{kl})\right)+\left(n+1\right)\partial_{i}\partial_{\gamma }\psi . \end{array}$$

Therefore, the formula $\partial_{a}{\overset{\left(\alpha \right)}{\Gamma }}_{bc}{}^{a}=\partial_{b}{\overset{\left(\alpha \right)}{\Gamma }}_{ac}{}^{a}$ holds when α=1 and $\overset{\left(1\right)}{\nabla }$ is equiaffine.

Second, we find the one-parallel prior. Let $\pi^{\left(1\right)}$ be the density of the one-parallel volume element. According to Proposition 1 in the paper by Takeuchi and Amari [17], we have

(69) $$\begin{array}{c} \partial_{a}\log\pi^{\left(1\right)}={\overset{\left(1\right)}{\Gamma }}_{ab}{}^{b}. \end{array}$$

In the case of the oTEF, its representation is

(70) $$\begin{array}{c} \partial_{i}\log\pi^{\left(1\right)}=\partial_{i}\log\left(-\partial_{\gamma }\psi \right),\partial_{\gamma }\log\pi^{\left(1\right)}=\partial_{\gamma }\log\left(-\partial_{\gamma }\psi \right). \end{array}$$

Therefore, the one-parallel prior for P is given as

(71) $$\begin{array}{cc} \pi^{\left(1\right)}\left(\theta ,\gamma \right) & \propto -\partial_{\gamma }\psi . \end{array}$$

□

Moreover, the above one-parallel prior coincides with a certain reference prior. A reference prior, proposed by Bernardo [24], is a noninformative prior distribution derived from an information-theoretic perspective. Specifically, it is defined as a prior distribution that maximizes the expectation of the KL divergence between the posterior and prior distributions, and in the case of no nuisance parameters, it coincides with the Jeffreys prior [24]. However, this is not necessarily the case when nuisance parameters are present. Ghosh and Mukerjee [25] provided a new formulation for reference priors with nuisance parameters by considering the maximization of a functional with an appropriate penalty term. Furthermore, Ghosal [26] extended this reference prior to non-regular models where the support of the density depends on the parameters. When applied to an oTEF model with γ as the parameter of interest and θ as the nuisance parameter, the reference prior of Ghosal [26] is given by

(72) $$\begin{array}{c} \pi_{\mathrm{Ghosal}}\left(\theta ,\gamma \right)\propto -\partial_{\gamma }\psi . \end{array}$$

Thus, it coincides with the one-parallel prior in Theorem 1.

## 5. Scalar Curvature on a Submodel of the oTEF

This section finds a submodel of the oTEF with constant scalar curvature for the Levi-Civita connection. We do not use the *α*-connection in Definition 8.

Previous works about the information geometry of non-regular cases have mainly studied the geometric structure of Pareto distributions for the Levi-Civita connection. They adopted the formally defined Fisher metric as in (42), which is consistent with our Riemannian metric. Rylov [10] found that the family of Pareto distributions has a constant curvature with respect to the Levi-Civita connection. Li et al. [12] showed that its geometrical structure is isometric to the Poincaré upper half-plane and applied this geometrical structure to Bayesian inference by considering the Jeffreys prior.

We extend these previous works on Pareto distributions to *n* dimensions. For i=1,…,n, let $F_{i}$ be a smooth function on the interval *I* and let $X_{i}$ be a random variable following a two-parameter truncated exponential distribution, which has density function

(73) $$\begin{array}{c} p_{E}\left(x,\theta^{i},\gamma \right)=\theta^{i}\exp\{-\theta^{i}\left(x-\gamma \right)\}\cdot {𝟙}_{\left[\gamma ,\infty \right)}\left(x\right)\;\left(x\in R\right) \end{array}$$

with respect to the Lebesgue measure, where $\theta^{i}\in \left(0,\infty \right),\;\gamma \in R$. An oTEF includes this distribution.

Consider $Q_{\theta ,\gamma }$, a joint distribution of independent random variables $F_{1}\left(X_{1}\right),\ldots ,F_{n}\left(X_{n}\right)$ with the common redefined truncation parameter γ and the natural parameter $\theta =(\theta^{1},\ldots ,\theta^{n})$. $Q_{\theta ,\gamma }$ has the density function

(74) $$\begin{array}{cc} q(x,\theta ,\gamma ) & =\exp\{-\sum_{i=1}^{n}\theta^{i}\left\{F_{i}\left(x_{i}\right)-F_{i}\left(\gamma \right)\right\}+\sum_{i=1}^{n}\log\theta^{i}\}\cdot {𝟙}_{I^{n}\left(\gamma \right)}\left(x\right) \end{array}$$

(75) $$\begin{array}{cc} \; & \left(x=\left(x_{1},\ldots ,x_{n}\right)\in I^{n}\right) \end{array}$$

with respect to the Lebesgue measure on $R^{n}$. Here, $I^{n}$ is a rectangle $\prod_{i=1}^{n}F_{i}\left(R\right)$ and I(γ) is $I^{n}\cap {\left[\gamma ,\infty \right)}^{n}$. Q is a family of distributions $Q_{\theta ,\gamma }$ with parameters θ and γ.

Q includes practical examples such as a family of several Pareto distributions with a common scale parameter (Rohatgi and Saleh [14]) and a family of truncated exponential distributions with a common location parameter (Ghosh and Razmpour [15]). Truncated exponential distributions with a common γ are sometimes applied to reliability and life testing. Please assume that the case where the first failure of *n* products can occur only after a common minimum time γ has elapsed, and these products have unknown and possibly unequal failure rates $\theta^{1},\ldots ,\theta^{n}$. This truncation parameter γ takes the role of a “guarantee time”, so estimation of the parameter γ is vital to determining the warranty period.

Note that the geometric structure of the above common truncation parameter model is equivalent to that of the family of Pareto distributions when n=1.

**Theorem** **2.** *Riemannian manifold Q has a constant scalar curvature of −2.*

**Proof.**  Let G(θ,γ) denote $\sum_{i}\theta^{i}F_{i}^{\prime }\left(\gamma \right)$, where $F^{\prime }\left(\gamma \right)=dF\left(\gamma \right)/d\gamma$.

The Riemannian metric for the common truncation parameter model is given by

(76) $$\begin{array}{cc} g_{ij} & =\frac{1}{{\left(\theta^{i}\right)}^{2}}\delta_{ij}, \end{array}$$

(77) $$\begin{array}{cc} g_{i\gamma } & =0, \end{array}$$

(78) $$\begin{array}{cc} g_{\gamma \gamma } & ={\left(G(\theta ,\gamma )\right)}^{2} \end{array}$$

for i,j=1,…,n. The tangent vectors $\partial_{1},\ldots ,\partial_{n},\partial_{\gamma }$ are mutually orthogonal. By (46), we have

(79) $$\begin{array}{cc} \Gamma_{ij,k} & =-\frac{1}{{\left(\theta^{i}\right)}^{3}}\delta_{ijk}, \end{array}$$

(80) $$\begin{array}{cc} \Gamma_{ij,\gamma } & =0, \end{array}$$

(81) $$\begin{array}{cc} \Gamma_{i\gamma ,j} & =0, \end{array}$$

(82) $$\begin{array}{cc} \Gamma_{i\gamma ,\gamma } & =F_{i}^{\prime }\left(\gamma \right)G\left(\theta ,\gamma \right), \end{array}$$

(83) $$\begin{array}{cc} \Gamma_{\gamma \gamma ,i} & =-F_{i}^{\prime }\left(\gamma \right)G\left(\theta ,\gamma \right), \end{array}$$

(84) $$\begin{array}{cc} \Gamma_{\gamma \gamma ,\gamma } & =G\left(\theta ,\gamma \right)\;\partial_{\gamma }G\left(\theta ,\gamma \right), \end{array}$$

and

(85) $$\begin{array}{cc} \Gamma_{ij}{}^{k} & =-\frac{1}{\theta^{i}}\delta_{ijk}, \end{array}$$

(86) $$\begin{array}{cc} \Gamma_{ij}{}^{\gamma } & =0, \end{array}$$

(87) $$\begin{array}{cc} \Gamma_{i\gamma }{}^{j} & =0, \end{array}$$

(88) $$\begin{array}{cc} \Gamma_{i\gamma }{}^{\gamma } & =\frac{F_{i}^{\prime }\left(\gamma \right)}{G(\theta ,\gamma )} \end{array}$$

(89) $$\begin{array}{cc} \; & =\partial_{i}\log G\left(\theta ,\gamma \right), \end{array}$$

(90) $$\begin{array}{cc} \Gamma_{\gamma \gamma }{}^{i} & =-\frac{F_{i}^{\prime }\left(\gamma \right)}{G(\theta ,\gamma )} \end{array}$$

(91) $$\begin{array}{cc} \; & =-\partial_{i}\log G\left(\theta ,\gamma \right), \end{array}$$

(92) $$\begin{array}{cc} \Gamma_{\gamma \gamma }{}^{\gamma } & =\frac{\partial_{\gamma }G\left(\theta ,\gamma \right)}{G(\theta ,\gamma )} \end{array}$$

(93) $$\begin{array}{cc} \; & =\partial_{\gamma }\log G\left(\theta ,\gamma \right) \end{array}$$

for i,j,k=1,…,n, where

(94) $$\begin{array}{c} \delta_{ijk}=\left\{\begin{array}{cc} 1 & \mathrm{for}\;\;i=j=k,\\ 0 & \mathrm{otherwise}. \end{array}\right. \end{array}$$

Hence, we obtain

(95) $$\begin{array}{cc} R_{i\gamma i}{\;}^{\gamma } & =\partial_{i}\Gamma_{\gamma i}{}^{\gamma }-\partial_{\gamma }\Gamma_{ii}{}^{\gamma }+\Gamma_{ia}{}^{\gamma }\Gamma_{\gamma i}{}^{a}-\Gamma_{\gamma a}{}^{\gamma }\Gamma_{ii}{}^{a} \end{array}$$

(96) $$\begin{array}{cc} \; & =\partial_{i}\partial_{i}\log G\left(\theta ,\gamma \right)+{\left(\partial_{i}\log G\left(\theta ,\gamma \right)\right)}^{2}+\frac{1}{\theta^{i}}\partial_{i}\log G\left(\theta ,\gamma \right) \end{array}$$

(97) $$\begin{array}{cc} \; & =-{\left(\frac{F_{i}^{\prime }\left(\gamma \right)}{G(\theta ,\gamma )}\right)}^{2}+{\left(\frac{F_{i}^{\prime }\left(\gamma \right)}{G(\theta ,\gamma )}\right)}^{2}+\frac{F_{i}^{\prime }\left(\gamma \right)}{\theta^{i}G\left(\theta ,\gamma \right)} \end{array}$$

(98) $$\begin{array}{cc} \; & =\frac{F_{i}^{\prime }\left(\gamma \right)}{\theta^{i}G\left(\theta ,\gamma \right)} \end{array}$$

for i=1,…,n.

Therefore, the scalar curvature is given by

(99) $$\begin{array}{cc} R & =R_{abcd}g^{ad}g^{bc} \end{array}$$

(100) $$\begin{array}{cc} \; & =-2. \end{array}$$

□

## 6. Concluding Remarks

This paper considered the geometric structure of a one-sided truncated exponential family (oTEF) with parameters θ and γ. We constructed a Riemannian metric based on the asymptotic properties of the maximum likelihood estimators. Under this, we showed that the formally defined α-connection admits α-parallel priors when α=1. Our result gives geometric meaning to a specific reference prior. Furthermore, we proved that the scalar curvature of some submodels of the oTEF obtained by making γ common across multiple distributions is constant.

It is essential to discuss suitable affine conditions for the oTEF. First, we need to reveal the statistical meaning of the α-connection in the oTEF. α-connection coefficients are expected to appear in higher-order terms of the variance of maximum likelihood estimators. Additionally, instead of the α-connection, we can construct a family of affine connections to be equiaffine by connecting the α-connection and the Levi-Civita connection. It is also interesting to consider affine connections induced by the third derivatives of divergences.

## Data Availability

Not applicable.
